# Efficacy of endoscopic radiofrequency ablation for proton pump inhibitor‐dependent gastroesophageal reflux disease: Multicenter prospective cohort study

**DOI:** 10.1111/den.14963

**Published:** 2024-12-04

**Authors:** Yuanxi Jiang, Zhiyu Dong, Ying Chen, Huihui Sun, Junwen Wang, Zhenxiang Wang, Qianqian Meng, Han Lin, Qingwei Zhang, Shengliang Chen, Zhizheng Ge, Luowei Wang, Shuchang Xu

**Affiliations:** ^1^ Department of Gastroenterology, Tongji Hospital，School of Medicine Tongji University Shanghai China; ^2^ Department of Gastroenterology, Digestive Endoscopy Center, Changhai Hospital Naval Medical University Shanghai China; ^3^ Key Laboratory of Gastroenterology and Hepatology, Division of Gastroenterology and Hepatology, Ministry of Health, Renji Hospital, School of Medicine, Shanghai Institute of Digestive Disease Shanghai Jiao Tong University Shanghai China

**Keywords:** dependent, endoscopic radiofrequency, gastroesophageal reflux disease, GERD‐HRQL, proton pump inhibitor

## Abstract

**Objectives:**

To evaluate the effects of endoscopic radiofrequency ablation (RFA) on proton pump inhibitor (PPI)‐dependent gastroesophageal reflux disease (GERD) in a Chinese population, and to explore the factors associated with favorable efficacy.

**Methods:**

A multicenter, single‐armed prospective cohort study was conducted. PPI‐dependent GERD patients were enrolled and underwent RFA. The primary outcome was improvement of GERD health‐related quality of life (GERD‐HRQL). Secondary outcomes were symptom improvement, satisfaction, PPI use, and the indicators of 24‐h pH‐impedance monitoring. A nomogram to predict complete remission was constructed.

**Results:**

In total, 66 patients were included. The GERD‐HRQL score was significantly reduced at the 3 month (mean difference, 14.7 [12.6–16.9]), 6 month (mean difference, 15.9 [13.8–18.1]), 12 month (mean difference, 16.7 [14.4–18.9]), 24 month (mean difference, 18.4 [16.2–20.1]), 36 month (mean difference, 18.2 [16.3–20.4]), and 48 month follow‐up (mean difference, 16.1 [14.2–18.3]), all *P* < 0.001. The esophageal and extra‐esophageal symptom scores were all significantly decreased. The proportion of satisfaction and no PPI use were significantly higher. With regard to the indicators of 24‐h pH‐impedance monitoring, acid exposure time (AET), and DeMeester score, but not lower esophageal sphincter (LES) pressure, decreased significantly at the 12 month follow‐up. A nomogram based on age, body mass index (BMI), baseline AET, and LES pressure was then constructed and showed good discrimination in the prediction of complete remission following RFA.

**Conclusions:**

This study demonstrated that RFA improved life quality as well as symptoms in PPI‐dependent GERD patients in a Chinese population. Younger age, higher BMI, lower baseline AET, and higher baseline LES pressure indicate favorable efficacy of RFA.

## INTRODUCTION

Proton pump inhibitors (PPIs) serve as the first‐line therapy of gastroesophageal reflux disease (GERD) management. However, most patients require long‐term use of PPIs.^1^, ^2^ Furthermore, the prolonged use of PPIs raises significant clinical side‐effects.^3^

Endoscopic radiofrequency ablation (RFA) of the lower esophageal sphincter (LES) has shown promising efficacy in PPI‐dependent GERD patients in Western countries.^4^, ^5^, ^6^, ^7^, ^8^, ^9^, ^10^, ^11^ However, few relevant studies have been conducted in China. Furthermore, few studies have focused on the factors associated with favorable efficacy of RFA. A predictive scoring system concentrating on complete remission following RFA in PPI‐dependent GERD patients is lacking, necessitating further investigation.

In this study, we aimed to evaluate the efficacy of RFA in PPI‐dependent GERD patients in a Chinese population. We also developed a nomogram model for clinical usage to predict complete remission in PPI‐dependent GERD patients following RFA.

## METHODS

### Study design

This was a multicenter, single‐armed prospective cohort study. Patients diagnosed with PPI‐dependent GERD were recruited in three tertiary general hospitals (Tongji Hospital Affiliated to Tongji University, the First Affiliated Hospital of Navy Military Medical University [Changhai Hospital], and Renji Hospital, Shanghai Jiao Tong University School of Medicine) from July 2017 to April 2022. This study was approved by the Ethics Committees of Tongji Hospital (Ethical batch number: [Tong] No. 375) and Changhai Hospital (Ethical batch number: CHEC2015‐086). Renji Hospital is one of the centers included in this study. However, as 24‐h pH‐impedance monitoring and esophageal high‐resolution manometry (HRM) instruments are not available in this hospital, participants meeting the inclusion criteria were transferred to Tongji Hospital for subsequent treatment, where informed consent was obtained, and included in the study. Therefore, Institutional Reviewer Board (IRB) approval was obtained in Tongji Hospital, and Renji Hospital contributed mainly to participant screening and enrolment.

### Patient selection criteria

The participants met the following criteria: (i) aged ≥18 years; (ii) met the diagnosis of GERD; and (iii) PPI treatment was effective but cannot be discontinued and the continuous use of PPIs has lasted for more than 6 months.

The exclusion criteria were as follows: (i) participants with hiatal hernia >2 cm; (ii) reflux esophagitis classified as Los Angeles grade C or D; (iii) previous esophageal or gastric surgery; (iv) esophageal motility disorders such as achalasia; (v) coagulation disorders; (vi) esophageal or gastric varices; (vii) cardiac pacemaker implantation; (viii) pregnancy.

GERD was diagnosed as follows: (i) the presence of GERD‐like symptoms including regurgitation, heartburn, chest pain, and so on; (ii) the presence of reflux esophagitis; or (iii) evidence of reflux on 24‐h pH‐impedance monitoring (acid exposure time [AET] >4%).

### Intervention

**Figure 1:**
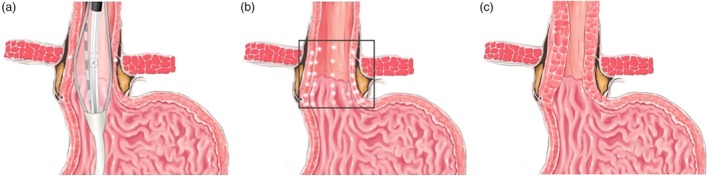
Endoluminal delivery of radiofrequency energy to the gastroesophageal junction. (a) Localization of balloon catheter at predetermined site in the gastroesophageal junction and delivered the radiofrequency energy for 1 min. (b) Radiofrequency ablation (RFA) thermal points within the gastroesophageal junction. A total of 80 puncture sites are usually associated with this therapy. (C) Schema showing “thickening” of the esophageal wall resulting from RFA therapy.

All participants underwent 24‐h pH‐impedance monitoring and esophageal HRM preoperatively, PPIs, H_2_ receptor antagonists, and prokinetics were discontinued for at least 1 week before gastroscopy or 24‐h pH‐impedance monitoring. A questionnaire was used to investigate the patients' symptoms, medication use, and satisfaction with life quality. The endoscopic RFA methods were described in our previous studies.^12^ RFA was performed using the RFA device, MER‐200GA (Medi Equip Medical Technology, Harbin, China), which has been approved by the China Food and Drug Administration and the National Medical Products Administration.^11^ During the sedated esophagogastroduodenoscopy (EGD), the endoscopists measured the distance to the gastroesophageal junction, and then introduced the radiofrequency delivery catheter orally. The endoscopists inflated the balloon 1.5 cm proximal to the gastroesophageal junction and delivered the radiofrequency energy for 1 min. The catheter was then rotated 45° and the procedure was repeated. This process was serially repeated every 0.5 cm, covering the area from 1.5 cm above to 1.5 cm below the gastroesophageal junction. The other six sets of this process were also used at the gastric cardia level and 0.5 cm above it, with the needle rotated 30° each time. Finally, a total of 80 lesions were treated at nine levels (Fig. 1).

### Outcome measures

The primary outcome measure was the GERD health‐related quality of life (GERD‐HRQL) score, which is currently the most commonly used scoring system for GERD patients.^13^

The secondary outcomes were esophageal and extraesophageal symptoms, satisfaction with life quality, PPI dosage, degree of reflux esophagitis, AET, DeMeester score, and LES pressure.

A nomogram model for clinical practice to predict complete remission (50% reduction in the GERD‐HRQL score and no PPI use at the 12 month follow‐up) was then developed based on the factors significantly associated with complete remission following RFA.

The symptom scores were collected by questionnaire using a 6‐point Likert scale. The frequency was graded as 0 (none), 1 (less than once a week), 2 (once or twice a week), 3 (three or four times a week), 4 (five or six times a week), and 5 (more than six times a week). The severity was graded as 0 (none), 1 (slight), 2 (mild), 3 (moderate), 4 (severe), and 5 (extremely severe). The sum of the frequency and severity score was designated as the score for each symptom.

### Follow‐up

**Figure 2:**
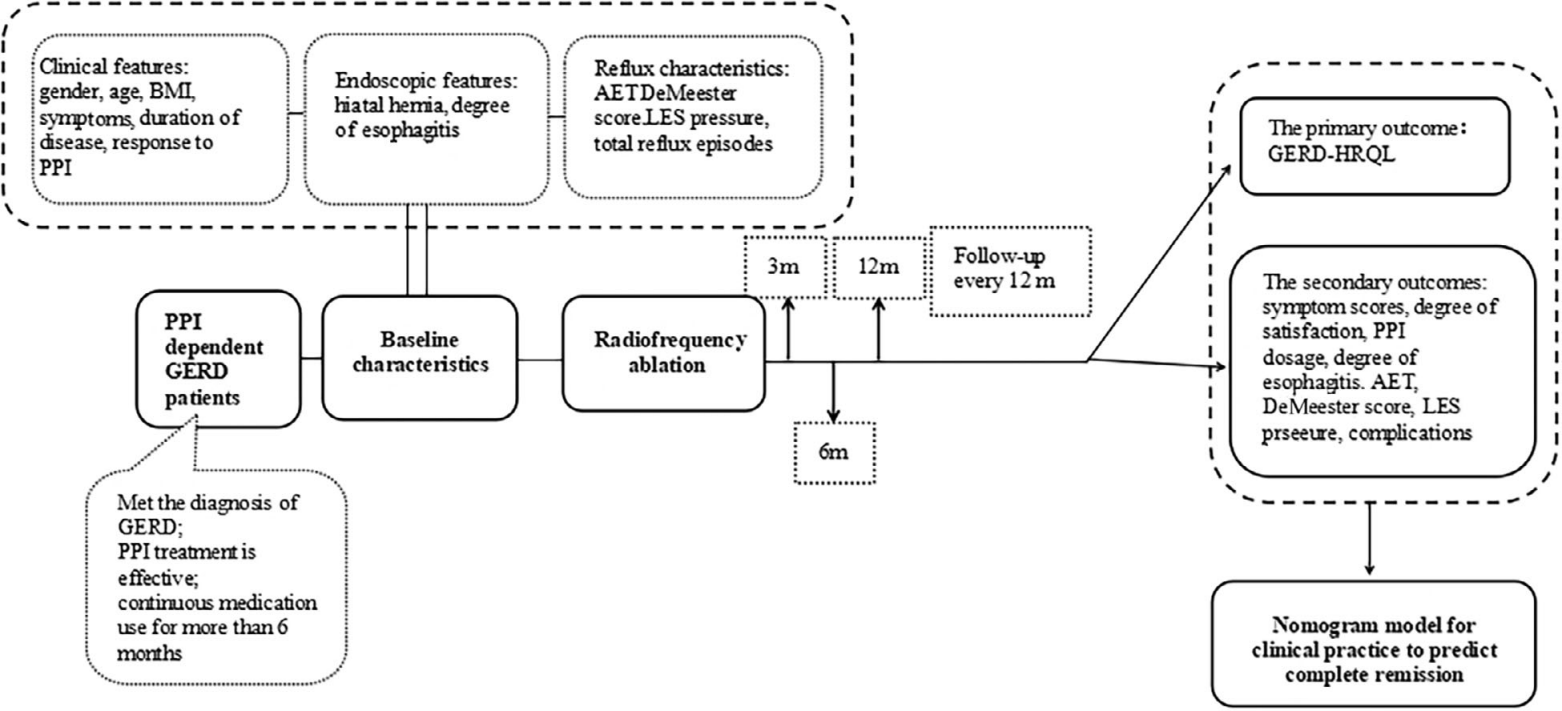
Study flow diagram. AET, acid exposure time; BMI, body mass index; GERD, gastroesophageal reflux disease; HRQL, health‐related quality of life; LES, lower esopheal sphincter; m, months; PPI, proton pump inhibitor.

Follow‐up was performed at 3, 6, 12, 24, 36, and 48 months following RFA, and the GERD‐HRQL score, symptom score, satisfaction of life quality, and PPI use were recorded. At 12 month follow‐up, 24‐h pH‐impedance monitoring, esophageal HRM and EGD were also performed (Fig. 2).

### Sample size calculations

Sample size calculations were performed using PASS 2021 software. According to previous studies, endoscopic RFA could decrease the GERD‐HRQL score by 10 points with a standard deviation (SD) of 10 in patients with PPI‐dependent GERD,^11^ setting *α* = 0.05 (two‐sided) and *β* = 0.1, and the calculated effective sample size was 11. If the number of subjects lost to follow‐up was not higher than 20%, at least 11 ÷ (1–20%) = 14 cases were enrolled.

### Statistical analysis

Participants with complete data at the 3, 6, 12, 24, 36, and 48 month follow‐up were included for final analysis. Data were analyzed using SPSS 22.0 software (IBM, Armonk, NY, USA). Continuous variables were expressed as mean ± SD for normally distributed variables or median (interquartile range) if the data were not normally distributed. Categorical variables were presented as number (*n*) and percentage (%). The paired Student's *t*‐test, repeated‐measure analysis of variance (ANOVA), χ^2^‐test, and Friedman test were used to compare the GERD‐HRQL score, symptom score, satisfaction of life quality, PPI use, degree of reflux esophagitis, the indicators of 24‐h pH‐impedance monitoring, and esophageal manometry. Multivariable logistic regression analysis was used to estimate adjusted odds ratio and 95% confidence interval for predicting complete remission following RFA. A nomogram based on significant variables was then constructed and the performance of discrimination was evaluated using receiver operating characteristic (ROC) curve and the area under the ROC curve (AUC).

All reported *P*‐values were two‐sided with *P* < 0.05 defined as statistically significant.

## RESULTS

**Figure 3:**
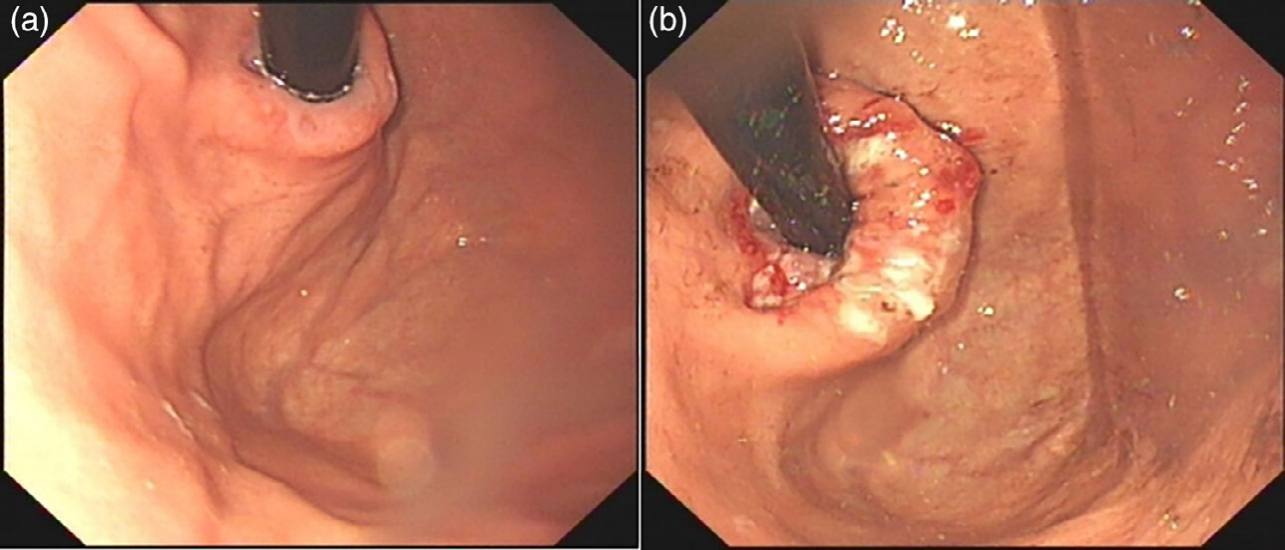
Endoscopic images (a) before and (b) after the procedure.

Three hundred and fifty‐three participants who had GERD‐like symptoms were enrolled; of these, 143 participants underwent 24‐h pH‐impedance monitoring and esophageal HRM. Of these patients, 85 had GERD, 33 had reflux hypersensitivity, and 25 had functional heartburn. The diagnosis of reflux hypersensitivity and functional heartburn was established according to Rome IV criteria. Finally, 70 patients agreed to participate in the study. Two patients were lost to follow‐up, one patient who was treated surgically for descending duodenal tumor during follow‐up, and one patient who underwent fundoplication 10 months after RFA due to unsatisfactory effect were excluded. In total, 66 participants were included in the final analysis and 31 participants underwent long‐term follow‐up (>12 months). There were 66, 66, 66, 31, 23, and 16 patients at the 3, 6, 12, 24, 36, and 48 month follow‐up. The baseline characteristics of these 66 patients are presented in Table 1. The average follow‐up time was 34.2 ± 10.8 months. The mean duration of the RFA procedure was 40.15 min, and the average hospitalization period was 2.3 days. The endoscopic image before and after the procedure are shown in Figure 3.

**Table 1:** Baseline characteristics of enrolled patients

Variable	Subjects (*n* = 66)
Age, years; mean (SD)	51.70 (14.23)
Female gender, *n* (%)	31 (47.0)
BMI, kg/m^2^; mean (SD)	23.40 (2.70)
Duration of disease, years; median [IQR]	3.00 [2.00, 5.00]
History of smoking, *n* (%)	58 (87.9)
History of alcohol intake, *n* (%)	53 (80.3)
Symptoms	
Heartburn, *n* (%)	46 (69.7)
Acid regurgitation, *n* (%)	55 (83.3)
Chest pain, *n* (%)	10 (15.2)
Pharyngalgia, *n* (%)	10 (15.2)
Cough, *n* (%)	10 (15.2)
GERD‐HRQL score, mean (SD)	24.39 (8.66)
24‐h pH‐impedance monitoring	
AET, median [IQR]	7.55 [5.03, 12.47]
DeMeester score, median [IQR]	26.28 [17.06, 43.26]
LES pressure, median [IQR]	12.45 [10.00, 18.15]

AET, acid exposure time; BMI, body mass index; GERD‐HRQL, gastroesophageal reflux disease health‐related quality of life; IQR, interquartile range; LES, low esophageal sphincter.

### Primary outcomes

**Figure 4:**
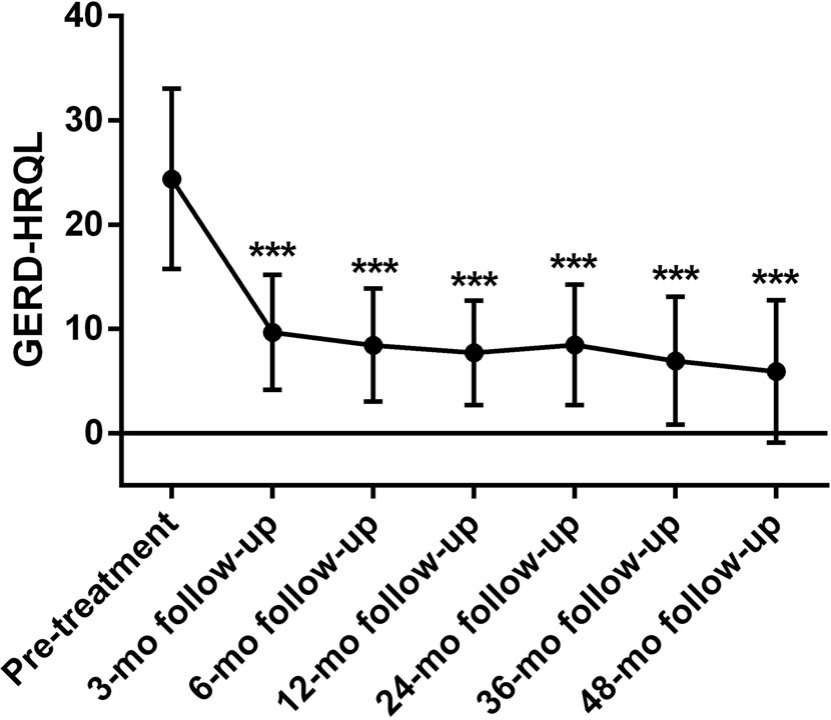
Gastroesophageal reflux disease health‐related quality of life (GERD‐HRQL) changes after radiofrequency ablation (RFA). Significant reductions in GERD‐HRQL scores at 3, 6, 12, 24, 36, and 48 month (mo) follow‐up following RFA. ***P < 0.001 when compared with baseline.

The GERD‐HRQL score significantly decreased by 14.7 ± 7.2, 15.9 ± 7.1, 16.7 ± 7.5, 18.4 ± 7.0, 18.2 ± 7.3, and 16.1 ± 7.5 at the 3, 6, 12, 24, 36, and 48 month follow‐up, respectively (all *P* < 0.001), suggesting that the life quality of PPI‐dependent GERD patients was significantly improved following RFA (Fig. 4).

### Secondary outcomes

#### Esophageal symptoms

**Figure 5:**
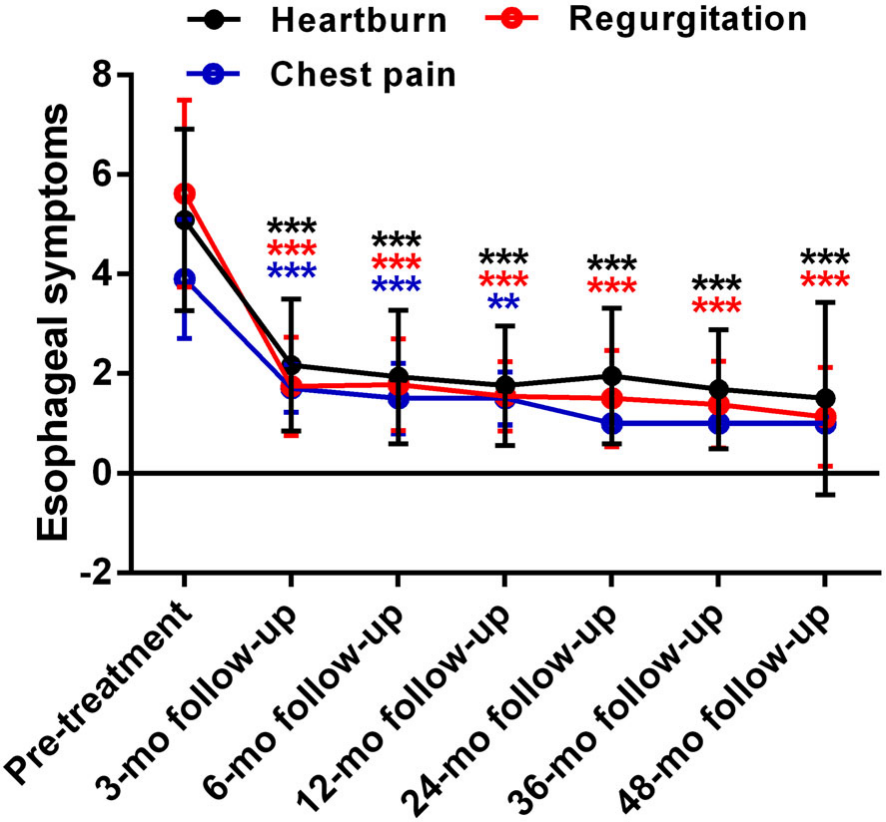
Esophageal symptoms changes after radiofrequency ablation (RFA). Significant reductions in esophageal symptom scores (heartburn, regurgitation, and chest pain) at 3, 6, 12, 24, 36, and 48 month (mo) follow‐up following RFA. **P < 0.01, ***P < 0.001 when compared with baseline.

RFA significantly improved heartburn, regurgitation, and the chest pain score (3 month follow‐up: mean decrease in heartburn, 2.9 ± 1.5; in regurgitation, 3.9 ± 1.8; in chest pain, 2.2 ± 1.1; all *P* < 0.001; 6 month follow‐up: mean decrease in heartburn, 3.2 ± 1.4; in regurgitation, 3.8 ± 1.6; in chest pain, 2.4 ± 1.1; all *P* < 0.001; 12 month follow‐up: mean decrease in heartburn, 3.3 ± 1.3; in regurgitation, 4.1 ± 1.7; in chest pain, 2.4 ± 1.3; all *P* < 0.001; 24 month follow‐up: mean decrease in heartburn, 3.8 ± 1.5, *P* < 0.001; in regurgitation, 4.0 ± 1.9, *P* < 0.001; in chest pain, 3.3 ± 0.6, *P* = 0.010; 36 month follow‐up: mean decrease in heartburn, 3.9 ± 1.7; in regurgitation, 4.1 ± 2.1, all *P* < 0.001; 48 month follow‐up: mean decrease in heartburn, 3.9 ± 2.0, *P* = 0.001; in regurgitation, 4.9 ± 1.8, *P* < 0.001. Almost all patients reported that chest pain had not reached their 36 month follow‐up at the time of analysis; Fig. 5).

#### Extraesophageal symptoms

**Figure 6:**
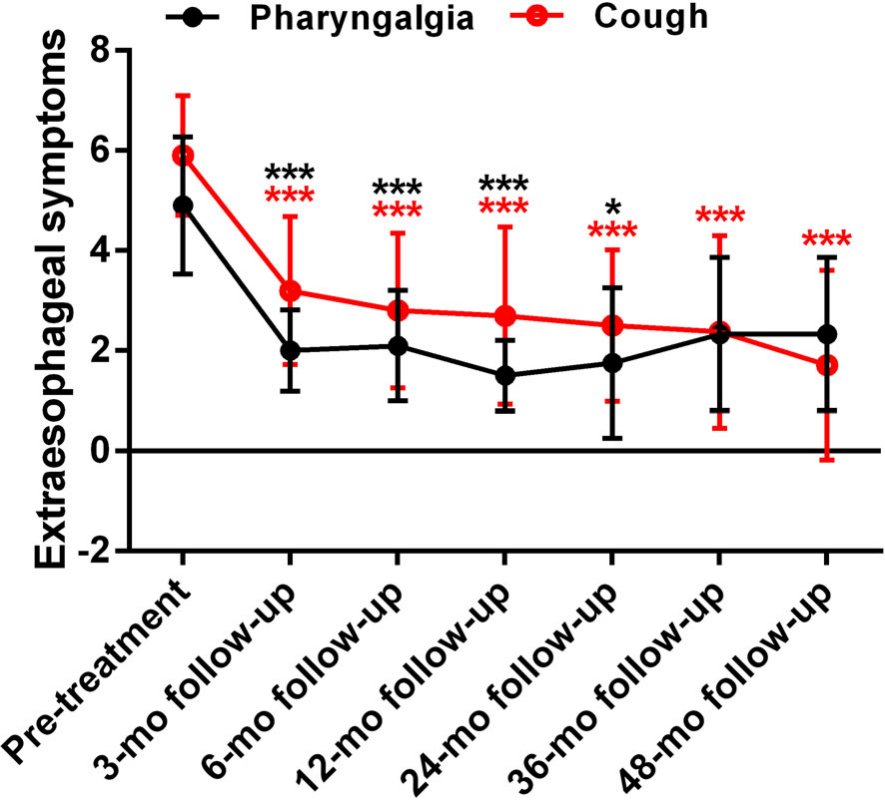
Extraesophageal symptoms changes after radiofrequency ablation (RFA). Significant reductions in extraesophageal symptom scores (cough and pharyngalgia) at 3, 6, 12, 24, 36, and 48 month (mo) follow‐up following RFA except pharyngalgia at 36 and 48 months. *P < 0.05, ***P < 0.001 when compared with baseline.

Cough and pharyngalgia scores were also improved at all follow‐up periods except pharyngalgia at 36 and 48 month (3 month follow‐up: mean decrease in cough, 2.7 ± 1.2; in pharyngalgia, 2.9 ± 1.0; all *P* < 0.001; 6 month follow‐up: mean decrease in cough, 3.1 ± 1.4; in pharyngalgia, 2.8 ± 1.1; all *P* < 0.001; 12 month follow‐up: mean decrease in cough, 3.2 ± 1.5; in pharyngalgia, 3.4 ± 1.2; all *P* < 0.001; 24 month follow‐up: mean decrease in cough, 3.2 ± 1.6, *P* = 0.001; in pharyngalgia, 4.0 ± 1.4, *P* = 0.011; 36 month follow‐up: mean decrease in cough, 3.4 ± 1.6, *P* = 0.001; in pharyngalgia, 4.0 ± 1.7, *P* = 0.057; 48 month follow‐up: mean decrease in cough, 4.0 ± 1.8, *P* = 0.001; in pharyngalgia, 4.0 ± 1.7, *P* = 0.057; Fig. 6).

#### Degree of satisfaction, PPI usage, and reflux esophagitis

**Figure 7:**
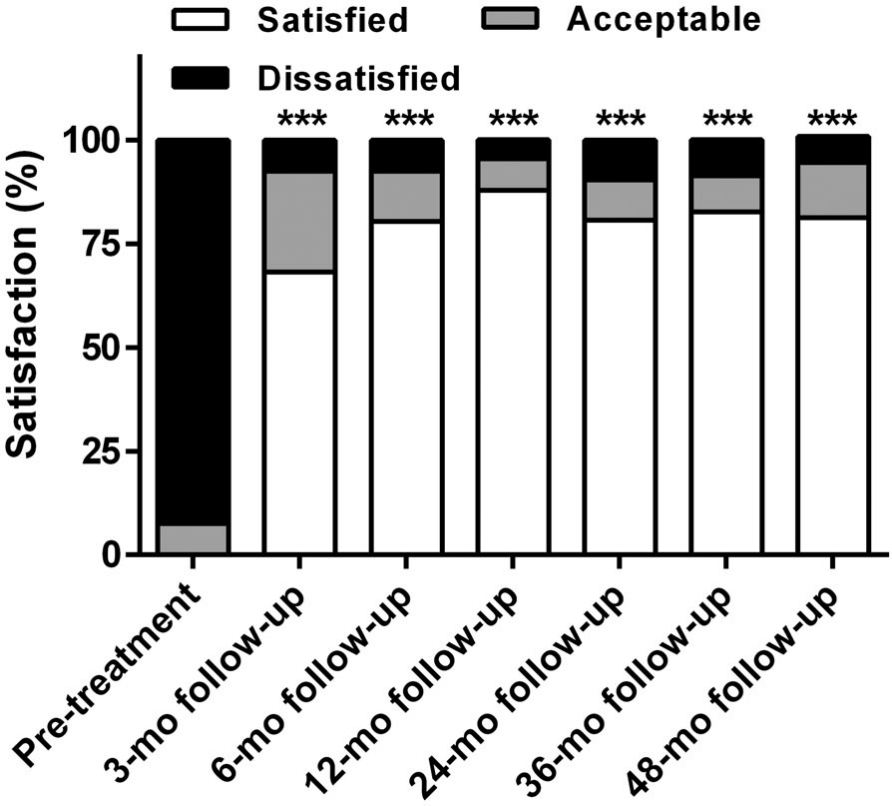
Degree of satisfaction with life quality before and after treatment. The radiofequency ablation significantly improved the degree of satisfaction with life quality at 3, 6, 12, 24, 36, and 48 month (mo) follow‐up. ***P < 0.001 when compared with baseline.

**Figure 8:**
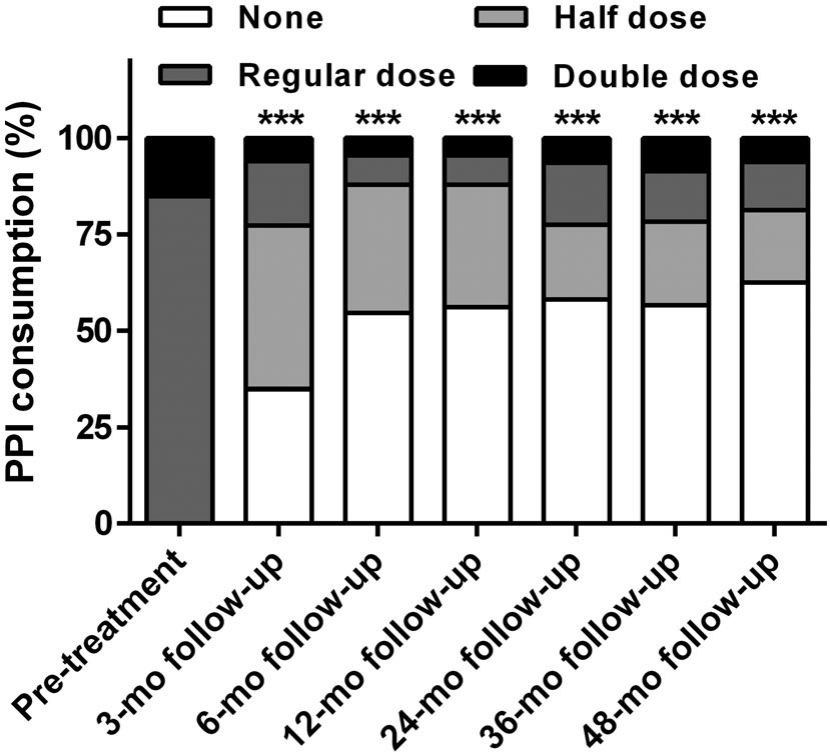
Degree of proton pump inhibitor (PPI) consumption before and after treatment. The radiofrequency ablation significantly reduced PPI usage at 3, 6, 12, 24, 36, and 48 month (mo) follow‐up. ***P < 0.001 when compared with baseline.

RFA significantly improved the degree of satisfaction and reduced PPI usage at all follow‐up periods (all *P* < 0.001). The rate of satisfaction increased to 68.2% (45/66), 80.3% (53/66), 87.9% (58/66), 80.6% (25/31), 82.6% (19/23), and 81.3% (13/16) at the 3, 6, 12, 24, 36, and 48 month follow‐up, respectively. PPI consumption was reduced in 84.8% (56/66), 90.9% (60/66), 90.9% (60/66), 83.9% (26/31), 82.6% (19/23), and 87.5% (14/16) of patients at the 3, 6, 12, 24, 36, and 48 month follow‐up, respectively. Of these, 34.8% (23/66), 54.5% (36/66), 56.1% (37/66), 58.1% (18/31), 56.5% (13/23), and 62.5% (10/16) patients stopped PPI use (Figs. 7,8).

**Figure 9:**
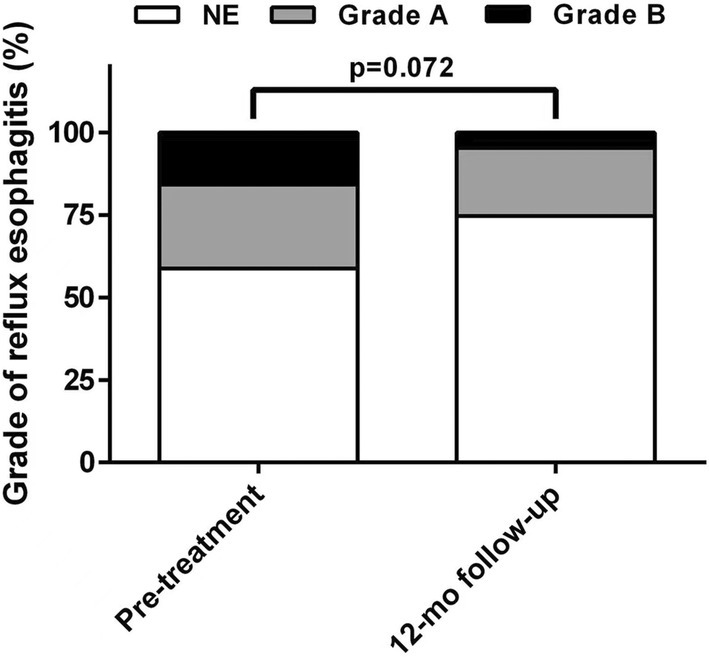
Grade of reflux esophagitis before and after treatment. No significant change in the severity grade with Los Angeles classification was observed before and after the radiofrequency ablation. NE, no esophagitis; mo, month.

Preoperatively, severity grade by the Los Angeles classification was: grade A, 24.2% (15/62); grade B, 16.1% (10/62); and no esophagitis, 59.7% (37/62). After RFA, grade A esophagitis, grade B esophagitis, and no esophagitis were observed in 19.4% (12/62), 4.8% (3/62), and 75.8% (47/62) of patients, which was not statistically significant compared with preoperatively (Fig. 9).

#### Indicators of pH‐impedance monitoring and esophageal manometry

**Figure 10:**
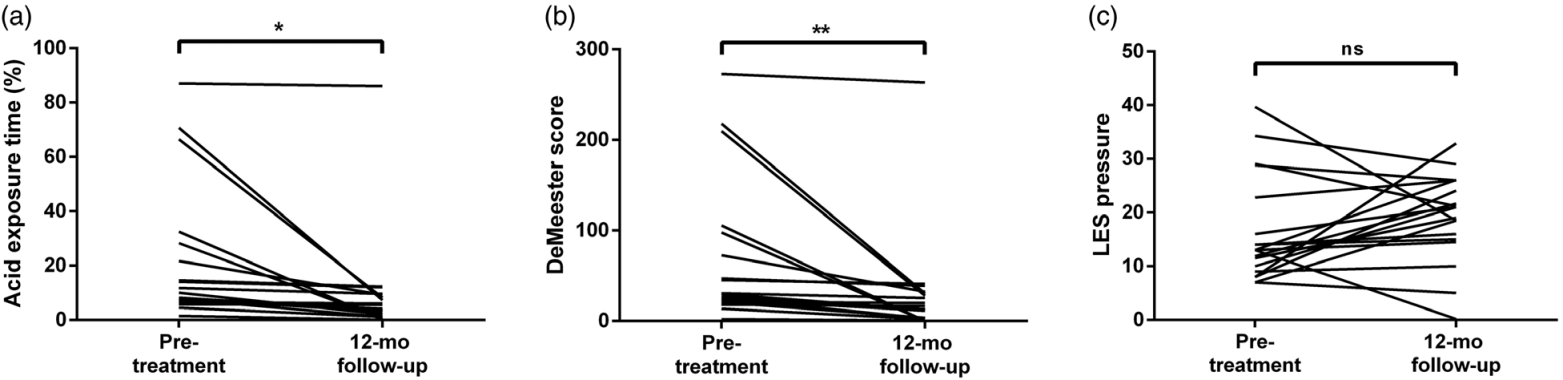
Indicators of pH‐impedance monitoring and esophageal manometry before and after treatment. (a) Acid exposure time and (b) DeMeester score, but not (c) lower esophageal sphincter (LES) pressure, were significantly lower than those before radiofrequency ablation. mo, month; ns, nonsignificance. *P < 0.05, **P < 0.01.

In our study protocol, 24‐h pH‐impedance monitoring and esophageal HRM were performed at baseline and 12 month follow‐up. However, the procedure is invasive and cannot be tolerated by some patients. Only 42 patients with complete baseline and 12 month follow‐up data were studied. The results showed that the AET and DeMeester score, but not LES pressure, were significantly lower than those before RFA (decrease in AET, 4.35 [2.43–7.63], *P* < 0.001; in DeMeester score, 15.9 [10.23–30.08], *P* < 0.001; in LES pressure, −1.45 [−4.85–0.68], *P* = 0.024; Fig. 10).

### Variables associated with complete remission following RFA

In order to explore which subtype of PPI‐dependent GERD patients may benefit more from RFA, a multivariate logistic regression model was used to identify the relationship between clinical characteristics at baseline and complete remission (50% reduction in GERD‐HRQL score and no PPI use) at the 12 month follow‐up after RFA. For each continuous variable, the cut‐off was identified using the best cut‐off to predict complete remission with the highest Youden's index. In multivariate regression analysis, age, body mass index (BMI), AET, and LES pressure were included in the final regression model, but only age <66.5 years, BMI ≥23.3, and LES pressure ≥16.25 were the independent indicators for complete remission following RFA (Table 2).

**Table 2:** Results of logistic regression model using complete remission as the outcome measure

	Univariate		Multivariate	
	OR (95% CI)	*P*‐value	OR (95% CI)	*P*‐value
Age	0.18 (0.04–0.68)	0.017	0.09 (0.01–0.52)	0.008
Female gender	1.31 (0.49–3.50)	0.589	–	–
BMI	2.65 (0.99–7.38)	0.055	6.14 (1.61–23.4)	0.008
Smoking	1.23 (0.27–5.67)	0.783	–	–
Drinking	0.70 (0.19–2.38)	0.573	–	–
Heartburn	0.73 (0.24–2.09)	0.558	–	–
Acid regurgitation	1.00 (0.26–3.70)	1.000	–	–
Chest pain	2.17 (0.54–10.9)	0.294	–	–
Pharyngalgia	2.17 (0.54–10.9)	0.294	–	–
Cough	0.30 (0.06–1.20)	0.103	–	–
AET	0.38 (0.13–1.05)	0.067	0.28 (0.08–1.04)	0.058
DeMeester score	0.48 (0.17–1.26)	0.140	–	–
LES pressure	4.47 (1.48–15.63)	0.011	6.41 (1.59–25.8)	0.009

AET, acid exposure time; BMI, body mass index; CI, confidence interval; LES, low esophageal sphincter; OR odds ratio.

### Nomogram based on age, BMI, baseline AET, and LES pressure to predict complete remission following RFA

**Figure 11:**
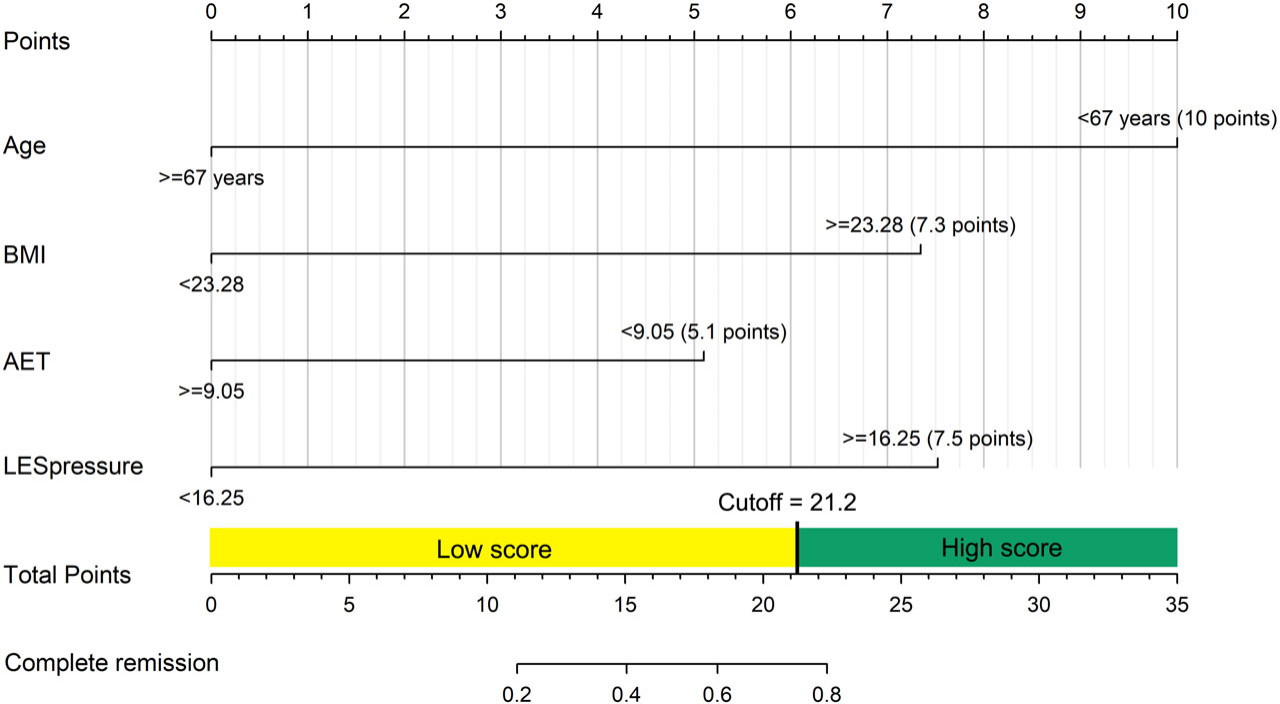
Nomogram based on the final multivariate logistic regression model to predict complete remission following radiofrequency ablation. Age, body mass index (BMI), acid exposure time (AET), and lower esophageal sphincter (LES) pressure were included in the final regression model.

**Figure 12:**
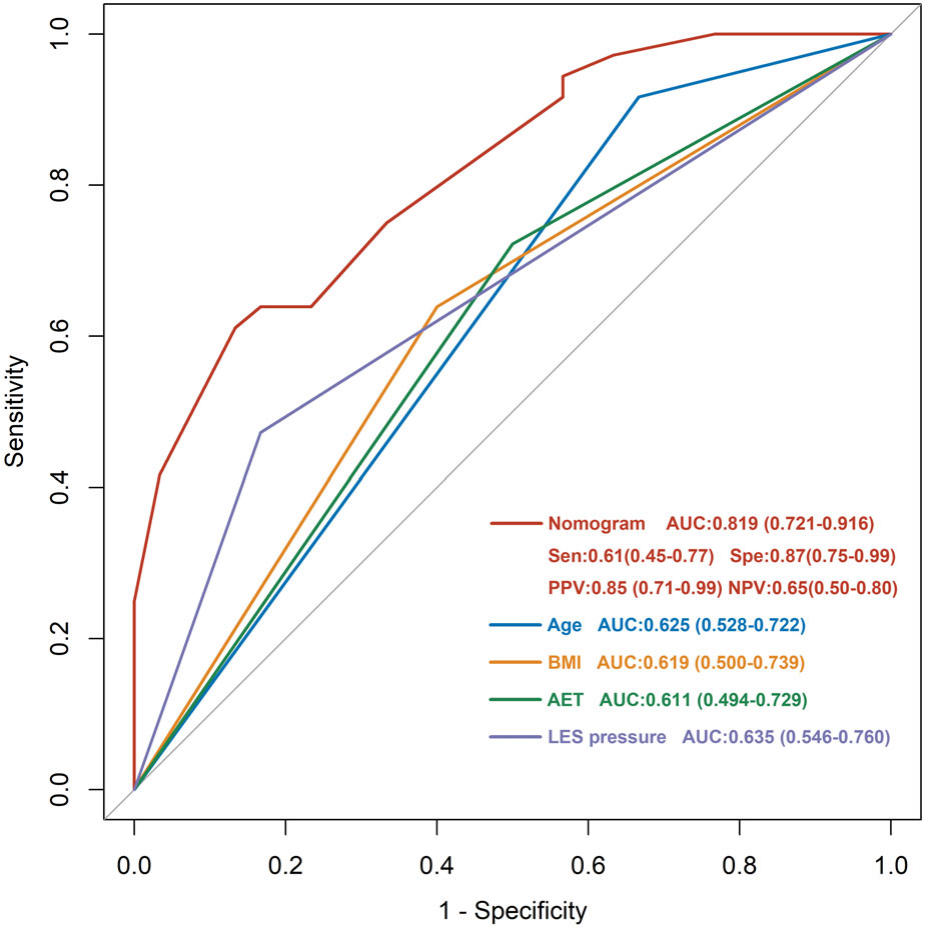
A nomogram based on age, body mass index (BMI), baseline acid exposure time (AET), and lower esophageal sphincter (LES) pressure to predict complete remission following radiofrequency ablation (RFA). The nomogram showed a good discrimination in prediction of complete remission following RFA (area under the curve [AUC] 0.819) and outperformed the predictive model using a single indicator (age, BMI, AET, and LES pressure). NPV, negative predictive value; PPV, positive predictive value; Sen, sensitivity; Spe, specificity.

A nomogram based on the final multivariate logistic regression model was developed to predict complete remission following RFA (Fig. 11). In this model, the sum of the point of each variable could be converted into the probability of complete remission. The cut‐off of this model was identified using the best cut‐off with the highest Youden's index (total points = 21.2, sensitivity = 0.61, specificity = 0.87, positive predictive value = 0.85, negative predictive value = 0.65, Youden's index = 0.48), which was used to divide the total points into the low‐score and high‐score group. The nomogram showed good discrimination in the prediction of complete remission following RFA (AUC 0.819) and outperformed the predictive model using single indicators (age, BMI, AET, and LES pressure; Fig. 12).

### Adverse events

Some patients experienced temporary minor adverse events, including sore throat (63.6%), transient nausea/vomiting (33.3%), retrosternal discomfort (27.3%), low‐grade fever (24.2%), belching (16.7%), transient dysphagia (12.1%), and abdominal distension (9.1%). In addition, one patient (1.5%) developed gastroparesis after treatment and was discharged from the hospital 10 days after rehospitalization. One patient (1.5%) developed upper gastrointestinal bleeding 7 days after treatment, and a review of gastroscopy showed one ulcer in the fundus; the patient recovered and was discharged 7 days after rehospitalization. No deaths occurred (Table 3).

**Table 3:** Adverse events after radio frequency ablation in enrolled patients

Adverse events, *n* (%)	Subjects (*n* = 66)
Perforation	0 (0.0)
Hematemesis	1 (1.5)
Gastroparesis	1 (1.5)
Sore throat	42 (63.6)
Retrosternal discomfort	18 (27.3)
Dysphagia	8 (12.1)
Fever	16 (24.2)
Nausea	22 (33.3)
Belching	11 (16.7)
Abdominal distension	6 (9.1)

## DISCUSSION

PPIs have been widely used for the management of GERD.^14^, ^15^, ^16^, ^17^, ^18^, ^19^ However, long‐term use of PPIs can greatly reduce life quality and lead to adverse reactions.^3^, ^4^ Previous studies have shown the promising efficacy of endoscopic RFA in GERD patients. However, there are few studies in China.^5^, ^6^, ^20^, ^21^ In the present study, the results showed that RFA significantly improved life quality by reducing both esophageal and extraesophageal symptoms. It also led to a reduction in PPI use and improved patients' satisfaction of life, which was similar to previous studies.^4^, ^5^, ^6^, ^22^, ^23^

There is a lack of consistency in the objective parameters (such as AET and LES pressure) of RFA in the current literature.^5^, ^6^, ^7^, ^9^, ^22^ In our study, AET and DeMeester score, but not LES pressure, were significantly lower than those at baseline, which was consistent with a recent meta‐analysis study.^23^ However, it is noteworthy that more than 30% of patients still had higher AET than normal after RFA, and LES pressure did not change significantly compared with that before RFA, indicating that the effect of RFA on local tissue structures may not fully explain the improvement of symptoms in patients.^24^, ^25^, ^26^

To the best of our knowledge, no previous studies have investigated the factors associated with the favorable efficacy of RFA and which subtype of PPI‐dependent patients may benefit more from this treatment. In our study, younger age, higher BMI, lower AET, and higher LES pressure at baseline were found to be associated with a higher likelihood of achieving complete remission. Joel *et al*.^27^ also found that RFA is effective for treating refractory GERD, especially in younger patients. The lower AET and higher LES pressure in GERD patients represented a lower prevalence of acid reflux and a more complete LES function, respectively. Thus, a better therapeutic effect of single RFA may be found in these patients. We also developed a nomogram based on these factors, including age, BMI, baseline AET, and LES pressure, which exhibited good discrimination in predicting complete remission following RFA. This nomogram has potential for future clinical practice. However, the predictive ability and generalization of this nomogram have not been validated in other large prospective cohorts and requires further exploration and validation.

In our study, RFA demonstrated a high level of safety. However, we observed one case of gastroparesis, with an incidence rate (1.5%) higher than that reported in previous studies,^23^, ^28^ suggesting that it is still necessary to comprehensively assess the risk–benefit ratio before RFA. Additionally, one patient experienced postoperative upper gastrointestinal bleeding and required rehospitalization. This adverse event may be attributed to the patient's failure to strictly follow postoperative instructions, indicating the significance of emphasizing perioperative management.

Several limitations in this study should be acknowledged. First, we were unable to include a sham‐controlled group due to study conditions. Additionally, as esophageal HRM and 24‐h pH‐impedance monitoring is invasive, some patients refused review after RFA, resulting in missing data and potentially impacting the assessment of the treatment effects to a certain extent.

In conclusion, our research confirmed the value of endoscopic RFA in the management of PPI‐dependent GERD with regard to improvement in quality of life, symptom relief, satisfaction, and PPI consumption. Younger age, higher BMI, lower AET, and higher LES pressure at baseline may predict better therapeutic effects.

## CONFLICT OF INTEREST

Authors declare no conflict of interest for this article.

## FUNDING INFORMATION

This work was supported by Shanghai Shenkang Hospital Development Center (grant numbers SHDC12016109), Shanghai Municipal Health Commission (grant numbers 202040079), Clinical Research Plan of SHDC (grant No. SHDC2022CRT004), “Science and Technology Innovation Action Plan” of STCSM (grant No. 22DZ2203900).

## ETHICS STATEMENT

Approval of the research protocol by an Institutional Reviewer Board: This study was approved by the Ethics Committees of Tongji Hospital (Ethical batch number: (Tong) No. 375) and Changhai Hospital (Ethical batch number: CHEC2015‐086). Renji Hospital is one of the centers included in this study. However, as 24‐h pH‐impedance monitoring and esophageal HRM instruments are not available in this hospital, participants meeting the inclusion criteria were transferred to Tongji Hospital for subsequent treatment, where informed consent was obtained, and included in the study. Therefore, IRB approval was obtained in Tongji Hospital, and Renji Hospital contributed mainly to participant screening and enrolment.

Informed Consent: All participants provided written informed consent.

Registry and the Registration No. of the study/trial: The trial was registered at ClinicalTrials.gov under identifier INR: 16009470.

Animal Studies: N/A.

## References

[1] Kalapala R , Karyampudi A , Nabi Z *et al*. Endoscopic full‐thickness plication for the treatment of PPI‐dependent GERD: Results from a randomised, sham controlled trial. Gut 2022; 71: 686–694. 33849942 10.1136/gutjnl-2020-321811 PMC8921577

[2] Patil G , Dalal A , Maydeo A . Feasibility and outcomes of anti‐reflux mucosectomy for proton pump inhibitor dependent gastroesophageal reflux disease: First Indian study (with video). Dig Endosc 2020; 32: 745–752. 31834663 10.1111/den.13606

[3] Noar M , Squires P , Noar E , Lee M . Long‐term maintenance effect of radiofrequency energy delivery for refractory GERD: A decade later. Surg Endosc 2014; 28: 2323–2333. 24562599 10.1007/s00464-014-3461-6

[4] Coron E , Sebille V , Cadiot G *et al*. Clinical trial: Radiofrequency energy delivery in proton pump inhibitor‐dependent gastro‐oesophageal reflux disease patients. Aliment Pharmacol Ther 2008; 28: 1147–1158. 18616516 10.1111/j.1365-2036.2008.03790.x

[5] Abdel Aziz AM , El‐Khayat HR , Sadek A *et al*. A prospective randomized trial of sham, single‐dose Stretta, and double‐dose Stretta for the treatment of gastroesophageal reflux disease. Surg Endosc 2009; 24: 818–825. 10.1007/s00464-009-0671-4 19730952

[6] Arts J , Bisschops R , Blondeau K *et al*. A double‐blind sham‐controlled study of the effect of radiofrequency energy on symptoms and distensibility of the gastro‐esophageal junction in GERD. Am J Gastroenterol 2012; 107: 222–230. 22108449 10.1038/ajg.2011.395

[7] Corley DA , Katz P , Wo JM *et al*. Improvement of gastroesophageal reflux symptoms after radiofrequency energy: A randomized, sham‐controlled trial. Gastroenterology 2003; 125: 668–676. 12949712 10.1016/s0016-5085(03)01052-7

[8] Dughera L , Navino M , Cassolino P *et al*. Long‐term results of radiofrequency energy delivery for the treatment of GERD: Results of a prospective 48‐month study. Diagn Ther Endosc 2011; 2011: 507157. 22110288 10.1155/2011/507157 PMC3202130

[9] Dughera L , Rotondano G , De Cento M , Cassolino P , Cisarò F . Durability of Stretta radiofrequency treatment for GERD: Results of an 8‐year follow‐up. Gastroenterol Res Pract 2014; 2014: 1–5. 10.1155/2014/531907 PMC4052191 24959175

[10] DiBaise JK , Brand RE , Quigley EMM . Endoluminal delivery of radiofrequency energy to the gastroesophageal junction in uncomplicated GERD: Efficacy and potential mechanism of action. Am J Gastroenterol 2002; 97: 833–842. 12003416 10.1111/j.1572-0241.2002.05597.x

[11] Liu PP , Meng QQ , Lin H *et al*. Radiofrequency ablation is safe and effective in the treatment of Chinese patients with gastroesophageal reflux disease: A single‐center prospective study. J Dig Dis 2019; 20: 229–234. 30873743 10.1111/1751-2980.12722

[12] Jiang YX , Dong ZY , Wang JW , Chen Y , Sun HH , Xu SC . Efficacy of endoscopic radiofrequency ablation for treatment of reflux hypersensitivity: A study based on Rome IV criteria. Gastroenterol Res Pract 2022; 2022: 4145810. 35386530 10.1155/2022/4145810 PMC8977342

[13] Velanovich V . The development of the GERD‐HRQL symptom severity instrument. Dis Esophagus 2007; 20: 130–134. 17439596 10.1111/j.1442-2050.2007.00658.x

[14] Spechler SJ . Refractory gastroesophageal reflux disease and functional heartburn. Gastrointest Endosc Clin N Am 2020; 30: 343–359. 32146950 10.1016/j.giec.2019.12.003

[15] Hsu PI , Lu CL , Wu DC *et al*. Eight weeks of esomeprazole therapy reduces symptom relapse, compared with 4 weeks, in patients with Los Angeles grade A or B erosive esophagitis. Clin Gastroenterol Hepatol 2015; 13: 859–866.e1. 25245625 10.1016/j.cgh.2014.09.033

[16] Fujimoto K , Hongo M . Risk factors for relapse of erosive GERD during long‐term maintenance treatment with proton pump inhibitor: A prospective multicenter study in Japan. J Gastroenterol 2010; 45: 1193–1200. 20607308 10.1007/s00535-010-0276-7

[17] Bayerdörffer E , Bigard MA , Weiss W *et al*. Randomized, multicenter study: On‐demand versus continuous maintenance treatment with esomeprazole in patients with non‐erosive gastroesophageal reflux disease. BMC Gastroenterol 2016; 16: 48. 27080034 10.1186/s12876-016-0448-x PMC4831110

[18] Khan Z , Alastal Y , Khan MA *et al*. On‐demand therapy with proton pump inhibitors for maintenance treatment of nonerosive reflux disease or mild erosive esophagitis: A systematic review and meta‐analysis. Gastroenterol Res Pract 2018; 2018: 6417526. 30158966 10.1155/2018/6417526 PMC6109549

[19] Goh KL , Benamouzig R , Sander P , Schwan T , EMANCIPATE . Efficacy of pantoprazole 20 mg daily compared with esomeprazole 20 mg daily in the maintenance of healed gastroesophageal reflux disease: A randomized, double‐blind comparative trial – the EMANCIPATE study. Eur J Gastroenterol Hepatol 2007; 19: 205–211. 17301646 10.1097/MEG.0b013e32801055d5

[20] Richards WO , Scholz S , Khaitan L , Sharp KW , Holzman MD . Initial experience with the Stretta procedure for the treatment of gastroesophageal reflux disease. J Laparoendosc Adv Surg Tech A 2001; 11: 267–273. 11642661 10.1089/109264201317054546

[21] Torquati A , Houston HL , Kaiser J , Holzman MD , Richards WO . Long‐term follow‐up study of the Stretta procedure for the treatment of gastroesophageal reflux disease. Surg Endosc 2004; 18: 1475–1479. 15791372 10.1007/s00464-003-9181-y

[22] Dundon JM , Davis SS , Hazey JW , Narula V , Muscarella P , Melvin WS . Radiofrequency energy delivery to the lower esophageal sphincter (S‐tretta procedure) does not provide long‐term symptom control. Surg Innovation 2008; 15: 297–301. 10.1177/1553350608324508 18829607

[23] Fass R , Cahn F , Scotti DJ , Gregory DA . Systematic review and meta‐analysis of controlled and prospective cohort efficacy studies of endoscopic radiofrequency for treatment of gastroesophageal reflux disease. Surg Endosc 2017; 31: 4865–4882. 28233093 10.1007/s00464-017-5431-2

[24] Kalapala R , Singla N , Reddy DN . Endoscopic management of gastroesophageal reflux disease: Panacea for proton pump inhibitors dependent/refractory patients. Dig Endosc 2021; 34: 687–699. 34651353 10.1111/den.14169

[25] Aggarwal P , Kamal AN . Reflux hypersensitivity: How to approach diagnosis and management. Curr Gastroenterol Rep 2020; 22: 42. 32651667 10.1007/s11894-020-00779-x

[26] Woodland P , Shen Ooi JL , Grassi F *et al*. Superficial esophageal mucosal afferent nerves may contribute to reflux hypersensitivity in nonerosive reflux disease. Gastroenterology 2017; 153: 1230–1239. 28734832 10.1053/j.gastro.2017.07.017

[27] Joel A , Konjengbam A , Viswanath Y *et al*. Endoscopic radiofrequency Stretta therapy reduces proton pump inhibitor dependency and the need for anti‐reflux surgery for refractory gastroesophageal reflux disease. Clin Endosc 2024; 57: 58–64. 37157958 10.5946/ce.2023.026 PMC10834287

[28] Pandolfino JE , Krishnan K . Do endoscopic antireflux procedures fit in the current treatment paradigm of gastroesophageal reflux disease? Clin Gastroenterol Hepatol 2014; 12: 544–554. 23811248 10.1016/j.cgh.2013.06.012 PMC3880639

